# Fenofibrate Intervention and Event Lowering in Diabetes (FIELD) study: baseline characteristics and short-term effects of fenofibrate [ISRCTN64783481]

**DOI:** 10.1186/1475-2840-4-13

**Published:** 2005-08-22

**Authors:** The FIELD Study Investigators

**Affiliations:** 1 The FIELD Study, C/o NHMRC Clinical Trials Centre, Mallett St Campus, University of Sydney NSW 2006, Australia

## Abstract

**Objective:**

The Fenofibrate Intervention and Event Lowering in Diabetes (FIELD) Study is examining the effects of long-term fibrate therapy on coronary heart disease (CHD) event rates in patients with diabetes mellitus. This article describes the trial's run-in phase and patients' baseline characteristics.

**Research design and methods:**

FIELD is a double-blind, placebo-controlled trial in 63 centres in 3 countries evaluating the effects of fenofibrate versus placebo on CHD morbidity and mortality in 9795 patients with type 2 diabetes mellitus. Patients were to have no indication for lipid-lowering therapy on randomization, but could start these or other drugs at any time after randomization. Follow-up in the study was to be for a median duration of not less than 5 years and until 500 major coronary events (fatal coronary heart disease plus nonfatal myocardial infarction) had occurred.

**Results:**

About 2100 patients (22%) had some manifestation of cardiovascular disease (CVD) at baseline and thus high risk status. Less than 25% of patients without CVD had a (UKPDS determined) calculated 5-year CHD risk of <5%, but nearly all had a 5-year stroke risk of <10%. Despite this, half of the cohort were obese (BMI > 30), most were men, two-thirds were aged over 60 years, and substantial proportions had NCEP ATP III features of the metabolic syndrome independent of their diabetes, including low HDL (60%), high blood pressure measurement or treatment for hypertension (84%), high waist measurement (68%), and raised triglycerides (52%).

After a 6-week run-in period before randomisation with all participants receiving 200 mg comicronized fenofibrate, there were declines in total and LDL cholesterol (10%) and triglycerides (26%) and an increase in HDL cholesterol (6.5%).

**Conclusion:**

The study will show the effect of PPAR-alpha agonist action on CHD and other vascular outcomes in patients with type 2 diabetes including substantial numbers with low to moderate CVD risk but with the various components of the metabolic syndrome. The main results of the study will be reported in late 2005.

## Introduction

The cardiovascular benefits of long-term treatment using HMG-CoA reductase inhibitors (statins) have been conclusively shown in several studies (summarized in [1]) of people with and without established cardiovascular disease, which have included more than 18000 men and women with diabetes mellitus [2-8]. To date, however, no clinical trials using peroxisome proliferator-activated receptor (PPAR) alpha agonists (such as fibrates) have specifically examined cardiovascular event rate changes in large numbers of patients with diabetes mellitus. In the 6 trials of fibrates reported to date the aggregate number of patients with diabetes mellitus is just over 2000 [9-14], with VA-HIT accounting for about 40% of these [11]. Fibrates cause favorable changes to the typical lipid profile of type 2 diabetes mellitus, by raising high-density lipoprotein (HDL) cholesterol, lowering triglycerides, and reversing the tendency to formation of small, dense, low-density lipoprotein (LDL) cholesterol particles [15-18].

If PPAR alpha agonists (and other modulators of the PPAR axis) are to assume a role as mainstream agents for reducing the risk of fatal and nonfatal cardiovascular events, adequately powered placebo-controlled trials, similar to the statin trials, are needed. They should include patients with diabetes of both sexes across a wide range of age and absolute cardiovascular risk.

The Fenofibrate Intervention and Event Lowering in Diabetes (FIELD) Study is a 3-country (Australia, New Zealand and Finland), 63-centre, double-blinded placebo-controlled trial evaluating the effects of fenofibrate compared with placebo on coronary heart disease morbidity and mortality in 9795 patients with type 2 diabetes [1]. The study incorporated a 6-week active-treatment run-in period, with all subjects receiving 200 mg comicronized fenofibrate before randomization to long-term placebo or active treatment. This article reports the baseline and treatment-entry characteristics of this cohort and defines the absolute risk of its study population.

## Methods

The design of the FIELD study has been described in detail elsewhere [1]. The study was approved by local ethics committees at each participating institution. In brief, FIELD is a randomized double-blind, placebo-controlled, parallel-group trial. Participants are middle-aged to elderly people (50 to 75 years of age) with type 2 diabetes mellitus considered to be at risk of coronary heart disease. The first patient in FIELD was registered in November 1997 and randomized in February 1998. A total of 14 247 subjects were registered, and 13 900 were screened in clinics for eligibility at Visit 1.

Patients were recruited from 63 centres in Australia, New Zealand and Finland. Those with and without known vascular disease were eligible for participation provided that, at randomization, the usual physician considered that there was no current indication for lipid-modifying treatment. This meant that some patients meeting the FIELD eligibility criteria for lipid levels were instead treated by their usual doctors once they became aware of the patients' lipid profile at screening; therefore, such patients were not randomized and were not included in the study. Patients with or without lipid abnormalities, such as low HDL cholesterol or elevated triglycerides, were eligible if the total blood cholesterol level at screening fell between 3.0 and 6.5 mmol/L (about 115–250 mg/dL) and either the total-to-HDL cholesterol ratio was 4.0 or higher, or the triglyceride level was over 1.0 mmol/L. Patients were excluded if they had triglyceride levels over 5.0 mmol/L. Lipid entry criteria were consistent with recruiting people who would not qualify for fully subsidised lipid-modifying treatment under the government guidelines in all 3 countries. Participants could not be taking any lipid-modifying therapy at the start of the dietary run-in period. However, the protocol allows for statin or other lipid-lowering therapy to be added at any time after randomization and recommends continuing study medication. The study is thus evaluating the role of fenofibrate on a background of usual care and will provide safety data on fibrate therapy in combination with other lipid-lowering treatments. A prespecified analysis will explore the effects of fenofibrate separately among those patients taking other lipid-lowering agents during follow-up and those taking study treatment alone. Intention-to-treat methods of analysis will result in conservative estimates of any effects of fenofibrate in the event that drop-ins to statin treatment are more frequent in the placebo-treated group. Confounding with respect to other drug use should be prevented by the randomization process, which will result in good balance across the treatment groups.

Patients known to have type 2 diabetes (identified through hospital clinic records, diabetes society membership lists and various patient registers) were invited to attend a special clinic. After clinical screening of patients for eligibility and obtaining informed consent, blood was taken for biochemical measurements of fasting glucose, lipids, apolipoproteins, fibrinogen, HbA1c, insulin, renal and liver biochemistry, and homocysteine, and urine was collected for albumin-to-creatinine ratio. The LDL cholesterol level was calculated from the Friedewald formula [19]. Biochemical analysis used standard methods in 2 central laboratories (Southpath, Flinders Medical Centre, Adelaide, Australia, and the National Public Health Institute, Helsinki, Finland) which were aligned by using reference material from the Canadian External Quality Assurance Laboratory, Vancouver, Canada, traceable back to CDC, Atlanta, USA. Both laboratories participated in local external quality assurance programs. Eligibility was confirmed for consenting participants. All screened patients were asked to follow agreed national dietary recommendations for type 2 diabetes mellitus and provided with literature summarizing key dietary recommendations. Patients were asked to return after 4 weeks of dietary adherence for review of their interest in participating in the long-term trial, and for a check of laboratory eligibility (triglyceride <5.0 mmol/L, serum creatinine <130 μmol/L, alanine aminotransferase (ALT) less than twice the upper limit of normal) and to commence a single-blind placebo run-in phase of one capsule daily with breakfast for a further 6-week period. Upon the patients' return after the placebo period, consenting eligible patients were then entered into a 6-week single-blind active run-in period. During this time, they were to take comicronized fenofibrate 200 mg once daily. The placebo run-period was conducted to familiarize patients with the long-term commitment to the trial. The active run-in was conducted to provide data on the short-term effects of fenofibrate therapy on cholesterol levels for all randomized patients.

After the 16-week run-in procedures had been completed, a further blood sample was taken at the time of randomization (week 0) to determine the short-term effects of the comicronized fenofibrate on total cholesterol, HDL cholesterol, triglycerides, LDL cholesterol and fibrinogen. At this visit, patients were randomized, by a stratified adaptive randomization scheme, to receive either fenofibrate (200 mg comicronized formulation) or matching long-term placebo as one capsule daily with breakfast [1]. The final patient was randomized to the study on 3 November 2000.

All patients were then followed up through regular visits to a special clinic set in place for the purposes of the study in addition to routine care provided through the family doctor and usual diabetes clinics.

For a primary outcome of CHD events (CHD death plus nonfatal MI), it is projected that approximately 500 CHD events will have occurred when a median of 5 years of follow-up has elapsed (during the first quarter of 2005); this represents an event rate of approximately 1% per annum. By this time the trial will have 80% power to detect an observed 22% reduction in CHD events (based on the intention-to-treat method of analysis). This will also provide 90% power to detect a 25% relative reduction in CHD events (based on intention-to-treat analysis). These calculations assumed an average drop-out rate from active treatment of 10% over the course of the study but allowed for a larger drop-in rate from placebo to open-cholesterol treatment of 17%* by the mid-point of the trial and 32% by study close, in view of the possible increased uptake of statin therapy after the Heart Protection Study.^7^ (*This was incorrectly referred to as 10% in the FIELD design paper,^1^ the assumption used in the original power calculations.)

Therefore, follow-up in the study, as stipulated in the protocol, was to be for a median duration of not less than 5 years and until 500 major coronary events had occurred, unless the study was terminated prematurely on the recommendation of the independent Safety and Data Monitoring Committee.

## Results

**Figure 1:**
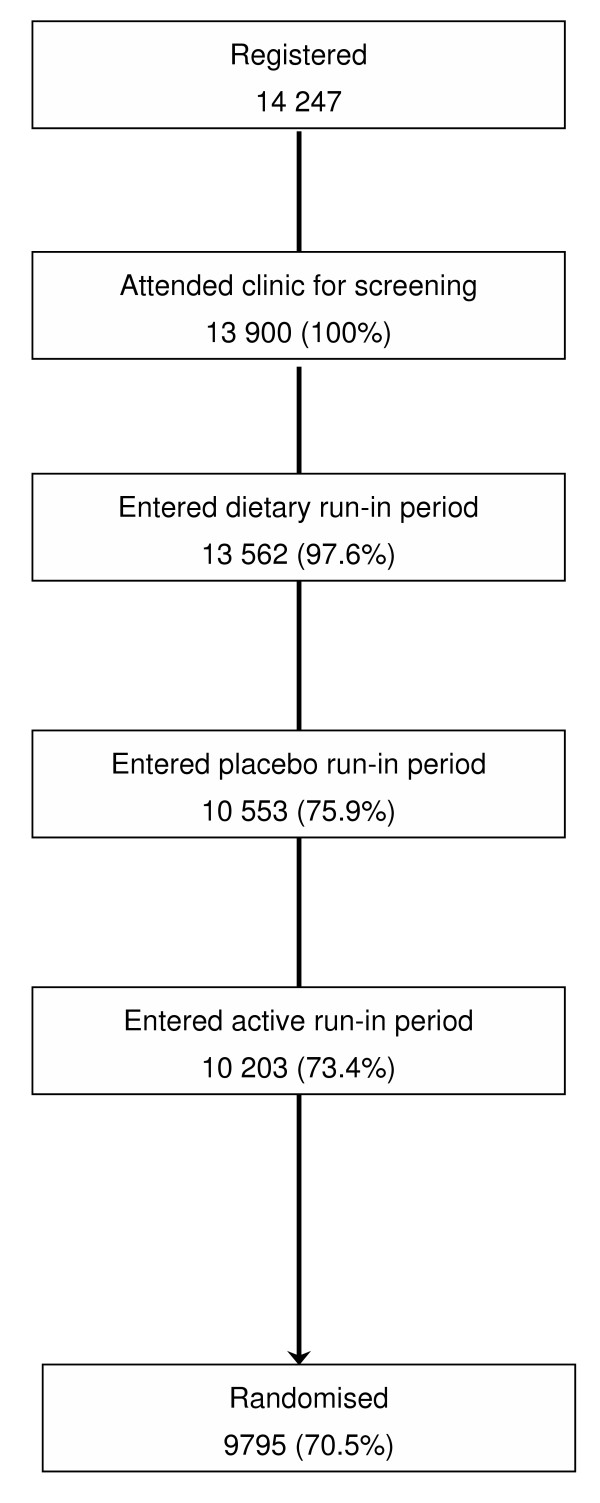
Numbers of patients enrolled in the run-in phases of the trial.

Of the 13 900 patients screened in study clinics, 75.9% (10 553) proceeded to enter the placebo run-in phase and 73.4% (10 203) the active run-in phase, and ultimately 9795 (70.5%) patients were randomized (Figure 1). The demographic characteristics are shown for the randomized and nonrandomized patients in Table 1. The study cohort included more men than women, and had a mean age in the mid-60s, and just over 2100 randomized patients had a prior history of cardiovascular disease.

**Table 1:** Baseline characteristics of the FIELD study cohort

**Characteristic**	**Category**	**Screened, not randomized** **(*n *= 4105)**		**Randomized** **(*n *= 9795)**	
		***n***	**%**	***n***	**%**
Sex	Male	2424	59.0	6138	62.7
	Female	1681	41.0	3657	37.3
Age at Visit 1 (years)	<55	682	16.6	1668	17.0
	55–59	802	19.5	1976	20.2
	60–64	856	20.8	2196	22.4
	65–69	888	21.7	2202	22.5
	70+	877	21.4	1753	17.9
Ethnicity*	Caucasian	3757	91.5	9093	92.8
	Indigenous	161	3.9	258	2.6
	Asian	93	2.3	141	1.4
	Other	94	2.3	303	3.1
Body mass index (kg/m^2^) (BMI)	BMI < 25	805	19.6	1198	12.2
	25 ≤ BMI < 30	1511	36.8	3855	39.4
	30 ≤ BMI < 35	1099	26.8	2869	29.3
	BMI ≥ 35	652	15.9	1866	19.1
Waist (cm)	Female (mean ± SD)	98 ± 15		101 ± 14	
	Male (mean ± SD)	103 ± 13		105 ± 12	
Waist-hip ratio	ratio < 0.8	242	5.9	333	3.4
	0.8 ≤ ratio < 0.9	1184	28.8	2531	25.8
	0.9 ≤ ratio < 1.0	1931	47.0	4942	50.5
	1.0 ≤ ratio < 1.1	645	15.7	1797	18.4
	ratio ≥ 1.1	73	1.8	179	1.8
Smoking (cigarettes)	Nonsmoker	1735	42.3	4000	40.8
	Ex-smoker	1933	47.1	4908	50.1
	Current smoker	404	9.8	887	9.1
Clinical history	Prior cardiovascular disease †	1045	25.4	2131	21.8
	Prior myocardial infarction	278	6.7	485	5.0
	Stroke	194	4.7	346	3.5
	Angina	588	14.3	1188	12.1
	Hypertension	2247	54.7	5544	56.6
	Claudication or peripheral vascular disease	351	8.6	711	7.2
	Transient ischemic attack	155	3.8	307	3.1
Prior coronary revascularisation	No CABG or PTCA	3908	95.2	9432	96.3
	CABG only	122	3.0	230	2.4
	PTCA only	49	1.2	102	1.0
	CABG and PTCA	26	0.6	31	0.3
Diabetes management	Diet only	1119	27.3	2542	26.0
	Diet + OH only	2238	54.5	5874	60.0
	Diet + insulin	429	10.5	569	5.8
	Diet + OH + insulin	319	7.8	810	8.3
Diabetic complications ‡	Retinopathy	431	10.5	814	8.3
	Neuropathy	636	15.5	1394	14.2
	Nephropathy	161	3.9	279	2.9
	Skin ulcers	139	3.4	299	3.1
	Amputations	106	2.6	176	1.8
Diabetes diagnosis ‡	Age at diagnosis (mean ± SD)	54.8 ± 8.7		55.5 ± 8.3	
	Median duration of diabetes in years (quartile 1, quartile 3)	6 (2, 11)		5 2, 10	

* Patients are assumed to be Caucasian unless otherwise stated. Indigenous includes Aborigines, Torres Strait Islanders, Maori and Pacific Islanders. Other includes mixed races, African-American, Indian, African, etc.

† Prior cardiovascular disease is defined as a reported history of myocardial infarction, angina (stable and unstable), coronary revascularization (CABG or PTCA), stroke, claudication or peripheral vascular disease or peripheral revascularization before randomization. The count includes 13 randomized patients and 10 nonrandomized patients who suffered or reported a cardiovascular event during the run-in period (visit 1 to visit 4 (randomization)).

‡ Diabetic complications and age at diabetes diagnosis are self-reported.

CABG = coronary artery bypass grafting; PTCA = percutaneous transluminal coronary angioplasty; SD = standard deviation; OH = oral hypoglycemic agents

There were 6051 subjects randomized from Australia, 2351 from New Zealand and 1393 from Finland. There were no differences across the 3 countries in mean ages of patients or ages at diagnosis (55.8 years, 53.0 years and 56.2 years for Australia, Finland and New Zealand, respectively). The median periods from diabetes diagnosis to randomization were 5, 7 and 5 years, respectively. The patients recruited were a mix from hospital and the community, and therefore were diverse in absolute risk. The 7664 participants who had no history of cardiovascular disease were at lower risk on the basis of interim determination using the modified Framingham equation of the UKPDS Risk Engine (Table 2) [20,21]. The prevalence rates of the various components of the metabolic syndrome (using criteria from NCEP ATP III definitions) at baseline are detailed in Table 3. A total of 84% of subjects met the ATP III criteria for metabolic syndrome. Baseline biochemistry for the entire randomized cohort and the effects of the 6 weeks of treatment with comicronized fenofibrate are shown in Table 4. On average, there was a 10% reduction in total cholesterol, a 10% reduction in LDL cholesterol, a 26% reduction in triglycerides and a 6.5% increase in HDL cholesterol after this short-term exposure to the active agent (average HDL increase was 6.1% in men versus 7.3% in women). Apolipoprotein B levels fell by an average of 14%, and the total-to-HDL cholesterol ratio by about 15%. Creatinine levels increased by an average of nearly 13% (an effect that is believed to be reversible and harmless, though not fully understood) [22,23], and fibrinogen levels fell by 11%.

**Table 2:** Distribution of projected 5-year risk (%, UKPDS risk engine) of coronary and other vascular outcomes for randomized patients with no history of cardiovascular disease (*n *= 7664)

**Outcome measures**	**Projected 5-year risk (%, from the UKPDS risk engine)**				
	**<5%**	**5%–<10%**	**10%–<15%**	**15%–<20%**	**≥20%**
All CHD	24.6	37.0	20.7	9.5	8.2
Fatal CHD	56.1	26.8	9.9	4.6	2.6
All stroke	79.3	17.0	2.8	0.7	0.2
Fatal stroke	99.9	0.1	0.0	0.0	0.0

**Table 3:** Prevalence of various components of the metabolic syndrome among the 9795 randomized participants in the FIELD study

	**Men** ** (*n *= 6138)**		**Women** ** (*n *= 3657)**		**Total** ** (*n *= 9795)**	
Metabolic syndrome feature: ATPIII criteria *	***n***	**%**	***n***	**%**	***n***	**%**
Waist measurement (women >88 cm; men >102 cm)	3613	58.9	3034	83.0	6647	67.9
Triglycerides >= 1.7 mmol/L	3073	50.1	2020	55.2	5093	52.0
HDL (men <40 mg/dL; women <50 mg/dL)	3365	54.8	2455	67.1	5820	59.4
Hypertension (SBP >= 130 and DBP >= 85 mm Hg)	5050	82.3	3131	85.6	8181	83.5
Fasting glucose >= 110 mg/dL or diabetes	6133	99.9	3646	99.7	9779	99.8

* When 3 of the 5 listed characteristics are present, a diagnosis of metabolic syndrome is established (84% of the cohort met the criteria)

**Table 4:** Baseline fasting biochemistry for the 9795 randomized participants in the FIELD study

	**Baseline**				**After 6 weeks of fenofibrate||**	
**Parameter**	**Mean**	**SD**	**Median**	**Q1, Q3**	**Mean**	**SD**
Total cholesterol (mmol/L)	5.04*	0.69	5.03*	4.56, 5.54	4.49	0.69
HDL cholesterol (mmol/L)¶	1.10*	0.26	1.06*	0.92, 1.24	1.16	0.29
Calculated LDL cholesterol (mmol/L)	3.07*	0.64	3.08*	2.62, 3.51	2.71	0.62
Triglycerides (mmol/L)	1.94*	0.88	1.74*	1.34, 2.34	1.37	0.63
Total-to-HDL cholesterol ratio	4.81*	1.10	4.71*	4.04, 5.49	4.08	1.13
Apolipoprotein B (g/L)	0.97*	0.17	0.97*	0.86, 1.09	0.83	0.18
Creatinine (mmol/L)	0.08†	0.02	0.08†	0.07, 0.09	0.09	0.02
Fibrinogen (g/L)	3.6†	0.8	3.6†	3.1, 4.1	3.12	0.75
Fasting glucose (mmol/L)	8.9†	2.6	8.5†	7.0, 10.3	--	--
Urinary albumin-to-creatinine ratio (mg/mmol)	5.9†	21.8	1.2†	0.7, 3.0	--	--
Homocysteine (μmol/L)	10.2§	3.7	9.5§	7.9, 11.6	--	--
Insulin (mU/L)	15.7‡	24.0	12.0‡	8.0, 18.5	--	--
HbA1c (%)	7.1†	1.4	6.9†	6.1, 7.8	--	--

* Average of Visit 2 and Visit 3.

† Average of Visit 1 and Visit 3.

‡ Visit 1 only.

§ Visit 3 only.

¶ Baseline HDLc (mean ± SD): men = 1.03 ± 0.23 mmol/L, women = 1.21 ± 0.28 mmol/L

|| All changes at six weeks were statistically significant at *P *< 0.0001

-- not measured

## Discussion

Almost all lipid-modifying trials that have included patients with diabetes have used statins as the intervention agent. Data on the effects of statin therapy among more than 18000 persons with type 2 diabetes are now available and show important reductions in coronary and vascular events [24] . Those studies involving more than 1000 people with diabetes include Anglo-Scandinavian Cardiac Outcomes Trial (ASCOT) [5], the Long-Term Intervention with Pravastatin in Ischaemic Disease (LIPID) study,[5] the Antihypertensive and Lipid-Lowering Treatment to Prevent Heart Attack Trial (ALLHAT-LLT) [4], the Heart Protection Study [7] and the most recently reported trial, the Collaborative Atorvastatin Diabetes Study (CARDS). CARDS used atorvastatin, 10 mg/day, and showed a 36% reduction in combined cardiovascular endpoints (2.46% per year for placebo and 1.54% for atorvastatin-treated) [8]. The results from a similarly structured trial (Atorvastatin Study for Prevention of Coronary Heart Disease Endpoints in Non Insulin Dependent Diabetes Mellitus, ASPEN) are pending.

Two large-scale trials of fibrate therapy have also been completed: the Veterans Low-HDL Cholesterol Intervention Trial (VA-HIT) and the Bezafibrate Infarct Prevention (BIP) trial enrolled subjects with and without diabetes mellitus. Both studies were limited to people with prior myocardial infarction and reported reductions in major cardiovascular events among participants with low HDL and high triglycerides at baseline, which were greater than with use of the same fibrate among those without dyslipidemia [11,25]. The VA-HIT trial also reported lower coronary heart disease mortality in those with diabetes receiving gemfibrozil and reduced cardiovascular events, though rates of nonfatal myocardial infarction did not change significantly [11]. A third, smaller, trial that used a fibrate, the Diabetes Atherosclerosis Intervention Study (DAIS), showed reduced progression of established coronary atherosclerosis among those randomized to fenofibrate compared with those receiving matching placebo over 3 years [10].

The identification of the PPAR transcription factor as the primary pathway through which fibrate and glitazone-agonist actions are triggered [18] has stimulated renewed interest in the antiatherogenic effects of these agents. PPAR-alpha activation by fibrates has the potential to prevent atherosclerosis via regulation of lipid metabolism and hemostatic pathways [26,27]. Consequently, there is great interest in the FIELD study because it will generate clinical information about fibrates in diabetes similar to that which is already available for the statins.

These baseline data of the FIELD study cohort without known prior cardiovascular disease show that over half such patients had a (UKPDS-determined) calculated 5-year coronary heart disease risk of less than 10% and nearly all patients had a stroke risk of less than 10% over 5 years. Part of the reason for this low-risk status may be that the duration of diabetes was only 5 years, on average, and reflecting this short duration, the median HbA1c was 6.9% for the entire cohort and the proportion of patients using no diabetes glucoregulatory therapy was just over a quarter. This probably reflects the recruitment process, in that many community-based subjects chose to enter the study in response to information provided by newsletters sent through Diabetes Australia, the Finnish Diabetes Society, the New Zealand Society for the Study of Diabetes and a New Zealand national diabetes consumer database.

Nonetheless, the cohort still had a profile with many characteristics of high cardiovascular risk. Half of the patients were obese (BMI > 30), most were male, two-thirds were over the age of 60 years, and substantial proportions had NCEP ATP III features of the metabolic syndrome additional to their diabetes mellitus, including low HDL cholesterol, either high blood pressure measurement and/or treatment for hypertension, high waist measurement and raised triglyceride. Over 2100 (22%) had established cardiovascular disease, and 39% of those without known cardiovascular disease had a projected 5-year absolute risk of a coronary event higher than 10%.

Evaluation of the effect of comicronized fenofibrate over 6 weeks immediately before randomization was included in the protocol. The purpose of this was to measure the effect of the agent in the entire cohort. After 6 weeks there were major reductions in triglycerides, lesser decreases in LDL cholesterol, and rises in HDL cholesterol. Creatinine levels rose while fibrinogen fell, several of the effects that have been observed elsewhere and with other fibrate agents [28,29].

Approximately 140 million adults were estimated to have diabetes mellitus in 1997; it is the most common endocrine disorder worldwide. Projections put diabetes prevalence by 2010 at 221 million, about 60 percent higher. Just as many people again have an elevated fasting glucose level, or impaired fasting glucose, which can progress rapidly to diabetes [30]. Without the FIELD study data, doctors would remain uncertain about the merits of using PPAR-alpha agonists, with or without concomitant statin therapy, when confronted with a patient with diabetes mellitus. The study will have special application to showing the effect of PPAR alpha agonist action on cardiovascular outcomes in those with low HDL dyslipidemic profiles. The main results of the FIELD study will be reported in late 2005.

## Declaration of competing interests

Of the Management Committee of the FIELD Study:

PB, YAK and RS have received reimbursements, fees, funding, or salary in the past five years from an organization that may in any way gain or lose financially from the publication of this paper;

No authors hold or have held stocks or shares in such an organization;

No authors have other financial competing interests;

AK has the following nonfinancial competing interest: Advisory board membership.

## Authors' contributions

The Writing Committee are the authors responsible for this article.

## Study organization

### Writing committee

R Scott, J Best, P Forder, M-R Taskinen, J Simes, P Barter, A Keech

### Management Committee

P Barter*, J Best*, P Colman, M d'Emden, T Davis, P Drury, C Ehnholm, P Glasziou, D Hunt, A Keech* (study chairman and principal investigator), YA Kesaniemi, M Laakso, R Scott*, RJ Simes*, D Sullivan, M-R Taskinen*, M Whiting; J-C Ansquer, B Fraitag (non-voting sponsor representatives). * Executive Committee members

### Outcomes Assessment Committee

N Anderson, G Hankey, D Hunt (chairman), S Lehto, S Mann, M Romo; LP Li (outcomes officer, in attendance),

### Safety and Data Monitoring Committee

C Hennekens, S MacMahon (chairman), S Pocock, A Tonkin, L Wilhelmsen; P Forder (unblinded statistician, in attendance).

### Site Principal investigators

***Australia: ***H Akauola, F Alford, P Barter, I Beinart, J Best, S Bohra, S Boyages, P Colman, H Connor, D Darnell, T Davis, P Davoren, F Lepre, F De Looze, M d'Emden, A Duffield, R Fassett, J Flack, G Fulcher, S Grant, S Hamwood, D Harmelin, R Jackson, W Jeffries, M Kamp, L Kritharides, L Mahar, V McCann, D McIntyre, R Moses, H Newnham, G Nicholson, R O'Brien, K Park, N Petrovsky, P Phillips, G Pinn, D Simmons, K Stanton, B Stuckey, D R Sullivan, M Suranyi, M Suthers, Y Tan, M Templer, D Topliss, J H Waites, G Watts, T Welborn, R Wyndham; ***Finland: ***H Haapamaki, A Kesaniemi, M Laakso, J Lahtela, H Levanen, J Saltevo, H Sodervik, M Taskinen, M Vanhala; ***New Zealand: ***J Baker, A Burton, P Dixon, J Doran, P Drury, P Dunn, N Graham, A Hamer, J Hedley, J Lloyd, P Manning, I McPherson, S Morris, C Renner, R Scott, R Smith, M Wackrow, S Young.

### Co-investigators and site coordinators

***Australia: ***F Alard, J Alcoe, F Alford, C Allan, J Amerena, R Anderson, N Arnold, T Arsov, D Ashby, C Atkinson, L Badhni, M Balme, D Barton, B Batrouney, C Beare, T Beattie, J Beggs, C Bendall, C Bendall, A Benz, A Bond, R Bradfield, J Bradshaw, S Brearley, D Bruce, J Burgess, J Butler, M Callary, J Campbell, K Chambers, J Chow, S Chow, K Ciszek, P Clifton, P Clifton-Bligh, V Clowes, P Coates, C Cocks, S Cole, D Colquhoun, M Correcha, B Costa, S Coverdale, M Croft, J Crowe, S Dal Sasso, W Davis, J Dunn, S Edwards, R Elder, S El-Kaissi, L Emery, M England, O Farouque, M Fernandez, B Fitzpatrick, N Francis, P Freeman, A Fuller, D Gale, V Gaylard, C Gillzan, C Glatthaar, J Goddard, V Grange, T Greenaway, J Griffin, A Grogan, S Guha, J Gustafson, P S Hamblin, T Hannay, C Hardie, A Harper, G Hartl, A Harvey, S Havlin, K Haworth, P Hay, L Hay, B Heenan, R Hesketh, A Heyworth, M Hines, G Hockings, A Hodge, L Hoffman, L Hoskin, M Howells, D Hunt, A Hunt, W Inder, W Inder, D Jackson, A Jovanovska, K Kearins, P Kee, J Keen, D Kilpatrick, J Kindellan, M Kingston-Ray, M Kotowicz, A Lassig, M Layton, S Lean, E Lim, F Long, L Lucas, D Ludeman, D Ludeman, C Ludeman-Robertson, M Lyall, L Lynch, C Maddison, B Malkus, A Marangou, F Margrie, K Matthiesson, J Matthiesson, S Maxwell, K McCarthy, A McElduff, H McKee, J McKenzie, K McLachan, P McNair, M Meischke, A Merkel, C Miller, B Morrison, A Morton, W Mossman, A Mowat, J Muecke, P Murie, S Murray, P Nadorp, S Nair, J Nairn, A Nankervis, K Narayan, N Nattrass, J Ngui, S Nicholls, V Nicholls, JA Nye, E Nye, D O'Neal, M O'Neill, S O'Rourke, J Pearse, C Pearson, J Phillips, L Pittis, D Playford, L Porter, L Porter, R Portley, M Powell, C Preston, S Pringle, W A Quinn, J Raffaele, G Ramnath, J Ramsden, D Richtsteiger, S Roffe, S Rosen, G Ross, Z Ross, J Rowe, D Rumble, S Ryan, J Sansom, C Seymour, E Shanahan, S Shelly, J Shepherd, G Sherman, R Siddall, D Silva, S Simmons, R Simpson, A Sinha, R Slobodniuk, M Smith, P Smith, S Smith, V Smith-Orr, J Snow, L Socha, T Stack, K Steed, K Steele, J Stephensen, P Stevens, G Stewart, R Stewart, C Strakosch, M Sullivan, S Sunder, J Sunderland, E Tapp, J Taylor, D Thorn, D Thorn, A Tolley, D Torpy, G Truran, F Turner, J Turner, J van de Velde, S Varley, J Wallace, J Walsh, J Walsh, J Walshe, G Ward, B Watson, J Watson, A Webb, F Werner, E White, A Whitehouse, N Whitehouse, S Wigg, J Wilkinson, E Wilmshurst, D Wilson, G Wittert, B Wong, M Wong, S Worboys, S Wright, S Wu, J Yarker, M Yeo, K Young, J Youssef, R Yuen, H Zeimer, R W Ziffer; ***Finland: ***A Aura, A Friman, J Hanninen, J Henell, N Hyvarinen, M Ikonen, A Itkonen, J Jappinen, A Jarva, T Jerkkola, V Jokinen, J Juutilainen, H Kahkonen, T Kangas, M Karttunen, P Kauranen, S Kortelainen, H Koukkunen, L Kumpulainen, T Laitinen, M Laitinen, S Lehto, R Lehto, E Leinonen, M Lindstron-Karjalainen, A Lumiaho, J Makela, K Makinen, L Mannermaa, T Mard, J Miettinen, V Naatti, S Paavola, N Parssinen, J Ripatti, S Ruotsalainen, A Salo, M Siiskonen, A Soppela, J Starck, I Suonranta, L Ukkola, K Valli, J Virolainen; ***New Zealand: ***P Allan, W Arnold, W Bagg, K Balfour, T Ball, B Ballantine, C Ballantyne, C Barker, C Barker, F Bartley, E Berry, G Braatvedt, A Campbell, T Clarke, R Clarke, A Claydon, S Clayton, P Cresswell, R Cutfield, J Daffurn, J Delahunt, A Dissnayake, C Eagleton, C Ferguson, C Florkowski, D Fry, P Giles, M Gluyas, C Grant, P Guile, M Guolo, P Hale, M Hammond, M Hammond, P Healy, M Hills, J Hinge, J Holland, B Hyne, A Ireland, A Johnstone, S Jones, G Kerr, K Kerr, M Khant, J Krebs, L Law, B Lydon, K MacAuley, R McEwan, P McGregor, B McLaren, L McLeod, J Medforth, R Miskimmin, J Moffat, M Pickup, C Prentice, M Rahman, E Reda, C Ross, A Ryalls, D Schmid, N Shergill, A Snaddon, H Snell, L Stevens, A Waterman, V Watts.

### Coordinating centre teams

***NHMRC Clinical Trials Centre, Sydney: ***K Jayne, E Keirnan, P Newman, G Ritchie, A Rosenfeld (project directors), E Beller, P Forder, V Gebski, A Pillai (study statisticians), C Anderson, S Blakesmith, S-Y Chan, S Czyniewski, A Dobbie, S Doshi, A Dupuy, S Eckermann, M Edwards, N Fields, K Flood, S Ford, C French, S Gillies, C Greig, M Groshens, J Gu, Y Guo, W Hague, S Healy, L Hones, Z Hossain, M Howlett, J Lee, L-P Li, T Matthews, J Micallef, A Martin, I Minns, A Nguyen, F Papuni, A Patel, J Pearse, R Pike, M Pena, K Pinto, D Schipp, J Schroeder, B Sim, C Sodhi, T Sourjina, C Sutton, R Taylor, P Vlagsma, S Walder, R Walker, W Wong, J Zhang, B Zhong, A Keech (deputy director), RJ Simes (director); ***Helsinki Project Office: ***A Kokkonen, P Narva, E-L Niemi, A Salo, A-M Syrjanen, M-R Taskinen (director); ***Christchurch Project Office: ***C Lintott, R Scott (director).

### Central laboratories

***Adelaide: ***R Tirimacco, M Whiting; ***Helsinki: ***C Ehnholm, M Ikonen, M Kajosaari, L Raman, J Sundvall, M Tukianen.

**Laboratoires Fournier SA liaison: ***Dijon: *J-C Ansquer, B Fraitag, D Crimet, I Sirugue, *Sydney: *P Aubonnet.
